# Flourishing Beyond Borders: Facilitating the Well-Being of Accompanying Expatriate Partners

**DOI:** 10.3389/fpsyg.2022.795845

**Published:** 2022-03-17

**Authors:** Truida Botha, Johan C. Potgieter, Karel F. H. Botha

**Affiliations:** ^1^Private Practitioner, Pretoria, South Africa; ^2^Department of Psychology, North-West University, Potchefstroom, South Africa

**Keywords:** accompanying expatriate partner, character strengths, expatriation, flourish, therapeutic techniques, resilience, well-being

## Abstract

One of the leading causes for failing at expatriate assignments is the accompanying expatriate partners’ (AEPs) unhappiness with life abroad or inability to adjust to the challenges of the host country. Strength-based therapeutic interventions have the potential to increase individuals’ mental health and well-being. The current study formed part of a multimethod study consisting of three related but independent sub-studies. The first sub-study identified the strengths of Gratitude, Curiosity and Hope to be positively associated with AEPs’ resilience and well-being. These results were used to construct a quantitative model that illustrates the interplay between these constructs. In the second sub-study, the proposed model was qualitatively reviewed by a smaller group of AEPs to inform and enrich our understanding of AEPs’ personal experiences of these constructs. In the current study, a panel of practicing psychologists who provide counselling services for South African expatriates and AEPs were asked to qualitatively review a proposed quantitative model. A cross-sectional, interpretive descriptive research design, applying purposive sampling was used to identify and recruit participants. The objective for the current study was firstly to ascertain why participants thought strengths of Gratitude, Curiosity and Hope featured so prominently in the model. Secondly, the study aimed to determine how these participants would, from their experience in working with AEPs, enhance these strengths and AEPs’ resilience in therapy, and ultimately facilitate greater well-being and successful adjustment abroad. Participants completed an online questionnaire consisting of two semi-structured, open-ended questions. The data were analyzed using primary and secondary cycle coding to ultimately develop themes. Results indicated that strengths of Curiosity, Gratitude and Hope featured prominently because these strengths include elements that form part of the process of expatriation. Participants were able to suggest practical strength-based therapeutic techniques which would assist in enhancement of strengths, resilience and ultimately well-being. It is proposed that the therapeutic techniques and approaches suggested in this study could contribute to the success rate of expatriate assignments.

## Introduction

While the expatriate employee deserves research attention, especially from a human resource perspective, it should be said that these employees are more often than not accompanied by their life partners and families. Harvey (1985) states that the employee’s family “has a profound impact on the success of the international assignment” (p. 84). Almost four decades later it is still the case, with an increasing focus on the role that so-called “accompanying expatriate partners” (AEPs), who may be a male or female, play in determining the success or failure of expatriate assignments (Lauring and Selmer, 2010; McNulty, 2012; Föbker and Imani, 2017; Lämsä et al., 2017). Numerous possible challenges that AEPs experience have been cited in recent research looking into the reasons for failed assignments. In most cases, the AEP has to give up a career in her^1^ home country “to follow the interests of her husband’s career” (Teague, 2015, p. 139). In addition, the AEP has to give up her home, social network and family in her home country, as well as establish a routine and find new pastime activities in the foreign country (Van den Berg-Overbreek, 2014; Lazarova et al., 2015; Teague, 2015). While the challenges faced by AEPs have received explicit research attention (Caligiuri and Bonache, 2016; Shortland, 2018; Arar, 2019; Harry et al., 2019), and the impact that AEPs have on the outcome of international assignments is a known fact, there has been a call for greater emphasis on protecting the mental health of AEPs (Wiese, 2013), especially through psychological interventions before international relocation.

It would therefore be in the interest of multinational companies (MNCs) and their human resource managers, as well as psychology practitioners, counsellors or business coaches working with AEPs, to understand what the positive predictors of the AEP’s well-being might be and how to facilitate the development of these predictors in AEPs’ lives. This could help equip AEPs with the necessary skills to confront the challenges that they might need to overcome in the host country in order to successfully adjust to life abroad (Naude and Vögel, 2018).

Although extensive research has been done on the pivotal role that AEPs play in the outcome of expatriate assignments, no study has been done where the enhancement of their character strengths, resilience and ultimately their well-being have been explored. This study aims to target this gap in the extant body of research.

## Materials and Methods

### Research Approach and Strategy

**Figure 1:**
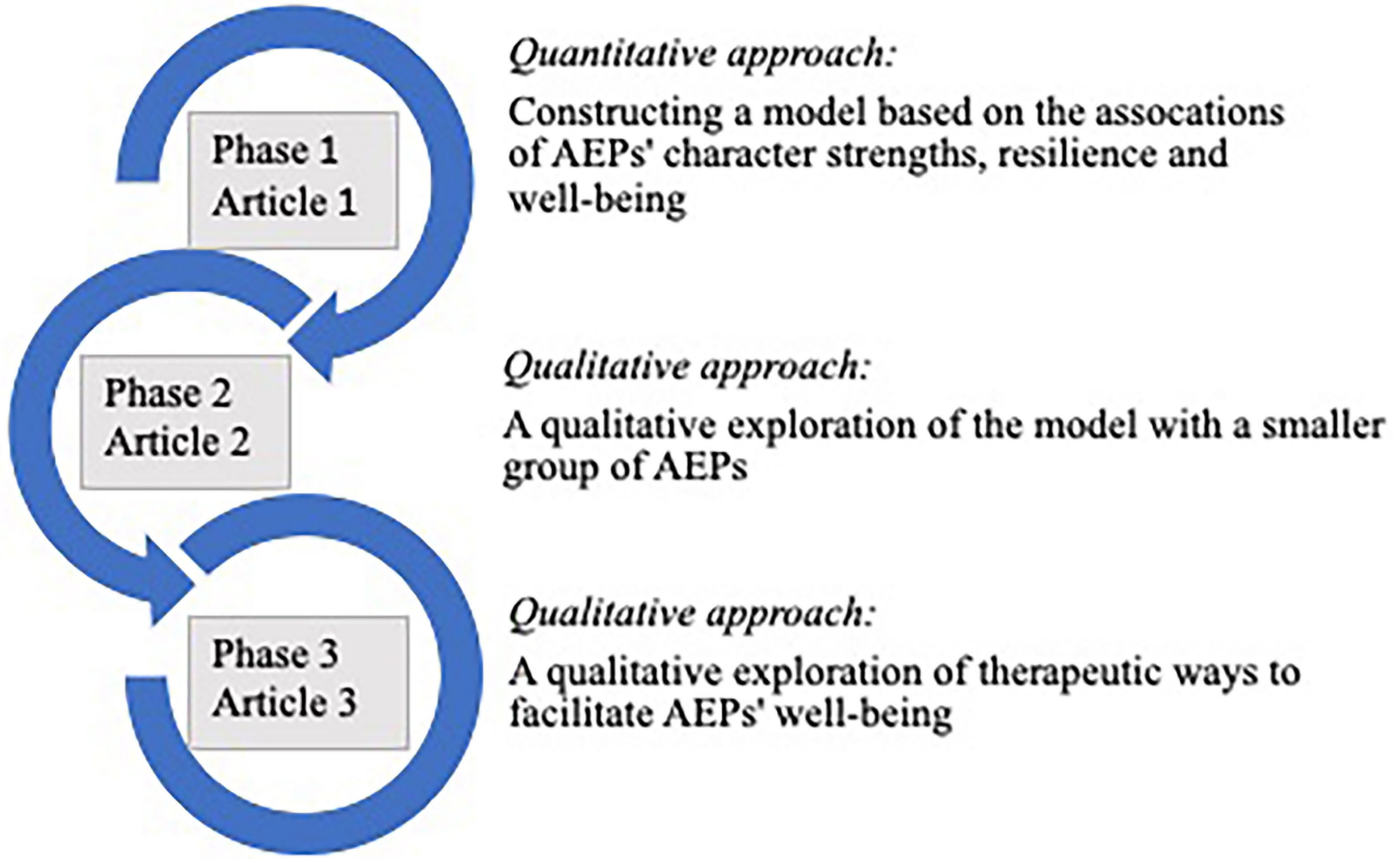
A graphical illustration of the multimethod research approach.

The current study forms part of a multimethod study (Morse, 2003) consisting of three related but independent phases of research that form part of a larger research project (Botha, 2020). These phases are graphically illustrated in Figure 1.

During the *first phase* of the study the character strengths, levels of resilience and self-perceived well-being of South African AEPs (*n* = 110) from 17 host countries were measured using the Virtues in Action Inventory of Strengths (*VIA*-72; Peterson and Seligman, 2004), the Resilience Scale (RS; Wagnild and Young, 1993) and the Mental Health Continuum – Short Form (MHC-SF; Keyes, 2002; Keyes et al., 2008). Structural equation modelling was used to construct a model indicating the associations between these constructs. During the *second phase* of the study, a smaller sample group (*n* = 17) representing seven of the original host countries was asked to qualitatively review the quantitative model that emerged during Phase 1, in an effort to gain greater understanding of their subjective experiences as AEPs and thereby enrich our understanding of the model.

**Figure 2:**
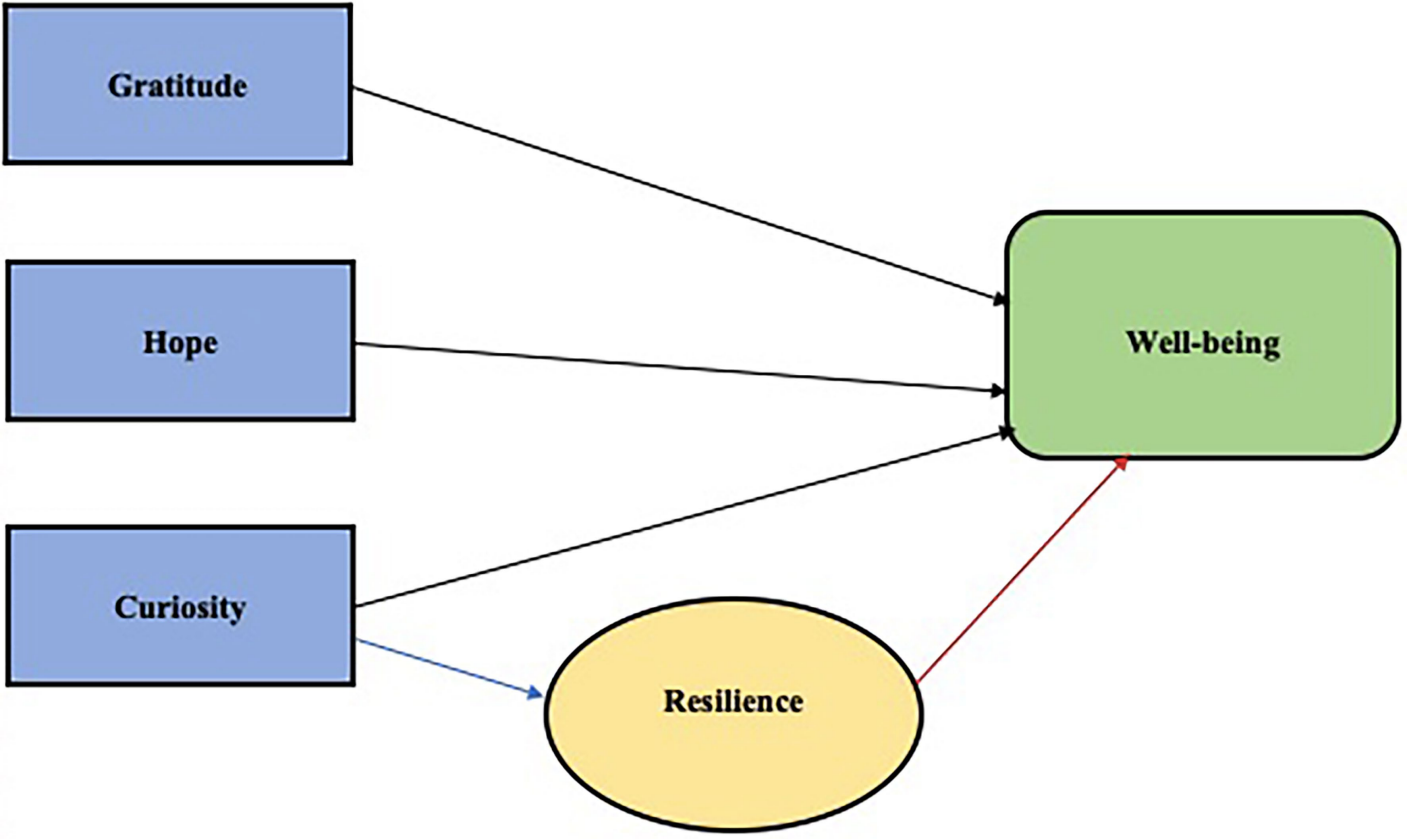
A graphical illustration of a simplified version of the reduced model presented to the panel of practicing psychologists.

The current study represents the *third and final phase* of the overall study where independent psychologists who provide counselling services for expatriates and AEPs were invited to share their thoughts and comments on the proposed model (see Figure 2). The objective was firstly to ascertain why these psychologists thought the strengths of Gratitude, Curiosity and Hope featured so prominently in the model. Secondly, the study aimed to determine how these psychologists would, from their experience in working with AEPs, enhance these strengths and AEPs’ resilience in therapy, and ultimately facilitate greater well-being and successful adjustment abroad. These results could potentially form the basis of an intervention framework for practitioners working with prospective or current AEPs. Since MNCs clearly have a vested interest in supporting not only the working partner (expatriate), but also the AEP and other family members, the insights and views expressed by experts in the field have the potential to effectively be used to increase the success rate of expatriate assignments.

### Entrée and Establishing Research Roles

Four psychologists were invited to participate in this phase of the study. The first author approached the participants by e-mail and explained the aims of the study, as well as what would be expected of them. Participation was completely voluntary and no costs were involved for the participants, nor were remuneration given. Due to the fact that the entire study was web-based, an informed consent document was sent to participants by e-mail. Informed consent was obtained from each participant before data were collected. The relevant background information regarding the previous phases of the study, as well as a simplified version of the reduced model (Figure 2), were shared with all the participants.

### Research Participants and Sampling Methods

Purposive sampling was used to recruit participants (Babbie, 2016). Participants were identified based on their knowledge and practical experience in working with expatriates, AEPs and their families. The four psychologists who participated were all registered with the Health Professions Council of South Africa (HPCSA), South African citizens and representative of three categories within psychology, namely industrial (*n* = 1; female), clinical (*n* = 1; female), and counselling (*n* = 2; 1 male and 1 female). Three of the psychologists were practicing in South Africa, and one psychologist was practicing abroad.

### Data Collection Methods

The entire study was web-based. A qualitative questionnaire containing two semi-structured, open-ended questions was used for data collection and sent to participants by e-mail.

With reference to the simplified version of the proposed quantitative model constructed based on the first two phases of the study (Figure 2), participants were asked the following questions:

- In your opinion and with your expertise, what might be possible explanations (or motivation) for Gratitude, Curiosity and Hope to be the three strengths that correlated positively with AEPs’ resilience and well-being, specifically bearing in mind the challenges faced by AEPs?
- If approached by an AEP/family about to embark on an expatriate assignment, how would you therapeutically address the enhancement of these character strengths and ultimately the resilience and well-being of AEPs? Please do not hesitate to be very practical in your reflections.

### Strategies Employed to Ensure Data Quality and Integrity

According to Hadi and Closs (2016), it is imperative for qualitative research findings to have *integrity.* The only way to ensure quality and integrity in qualitative data is by demonstrating *rigor*. Noble and Smith (2015) concur, and furthermore state that research integrity is essential if findings “are to be utilized in practice” (p. 34). The researchers employed a number of strategies to ensure data quality and integrity in this study. *Validity* was ensured by using purposeful sampling to meet the aims of the study (Smith and Noble, 2014) and continuous checking of data, coding of data, and interpretation of findings. The researchers furthermore used continuous self-reflection by keeping a reflective journal (Long and Johnson, 2000; Morse, 2015; Hadi and Closs, 2016); making code, theoretical and operational notes (Babbie, 2016); and acknowledging possible biases in the research process (Smith and Noble, 2014). *Reliability* was ensured by keeping an audit trail (Morse et al., 2002; Morse, 2015; Hadi and Closs, 2016) and appointing an external code reviewer to reduce research bias and to confirm the dependability of research findings. According to Noble and Smith (2015), an “independent researcher should be able to arrive at similar or comparable findings” (p. 34) for reliability of findings to be confirmed. In the current study, the data were coded by two independent experts in qualitative research, where after the coding, notes, analysis and interpretation were compared.

### Data Analysis

The data were analyzed using two phases (primary and secondary cycle coding) to develop themes (Tracy, 2013; Saldaña, 2016; Creswell and Poth, 2018). First cycle coding included open-coding and descriptive coding to explore and explain psychologists’ descriptions of the connection between strengths of Hope, Gratitude and Curiosity with resilience and well-being of AEPs. Second cycle coding entailed conceptual level analysis to consider the relationship between the different therapeutic approaches on which psychologists rely to enhance AEPs’ character strengths, resilience and ultimately their well-being. In order to ensure rigorous, systematic analysis, the coding techniques and principles of Saldaña (2016) were applied. Coding was facilitated using ATLAS.ti (Version 8; Friese, 2019).

### Reporting Style

In order to honor participants’ anonymity and ensure that their responses were dealt with in a confidential manner, no identifying elements were included in the data and the researchers’ notes were stored securely. Psychologists who participated in the study were identified as “Participant 1″, “Participant 2″ etc. The participants’ categories of registration in psychology were as follows: Participant 1 (counselling; female), Participant 2 (clinical; female), Participant 3 (counselling; male), and Participant 4 (industrial; female).

### Ethical Clearance

Ethical approval was obtained from the Health Research Ethics Committee (HREC) of the NWU (ethics approval number NWU-00362-16-A1). Throughout the entirety of the study, the researcher adhered to the ethical guidelines of the North-West University’s Health Research Ethics Committee (NWU-HREC) as well as the Health Professions Council of South Africa (HPCSA: Health Professions Act 56 of 1974).

## Results

Four distinct themes and eight sub-themes emerged from the participants’ perspectives on the role that these character strengths play on AEPs’ resilience and well-being abroad, as well as therapeutic approaches or techniques that could enhance these constructs. The main themes were labeled: (1) Perspectives on Curiosity, Gratitude and Hope; (2) The positive impact of strengths; (3) Therapeutic engagement and goal-setting; and (4) Facilitating well-being through strengths and resilience. The sub-themes were labeled: (1a) Elements of strengths and (1b) The time frame of strengths; (2a) Positive experiences of strengths and (2b) Personal growth; (3a) Assessment of context and (3b) Setting goals; and (4a) Cultivating strengths and resilience and (4b) Organizational support and other supportive relationships.

The themes and sub-themes are graphically illustrated in Table 1 and described below, with supportive evidence cited from the data transcripts (quotation references in parenthesis, e.g., Participant number; Quote number).

**Table 1:** Themes and sub-themes.

Themes	Sub-themes
1. Perspectives on Curiosity, Gratitude and Hope	Elements of strengths
	The time frame of strengths
2. The positive impact of strengths	Positive experiences of strengths
	Personal growth
3. Therapeutic engagement and goal-setting	Assessment of context
	Setting goals
4. Facilitating AEP’s well-being through strengths and resilience	Cultivating strengths and resilience
	Organizational support and other supportive relationships


### Perspectives on Curiosity, Gratitude and Hope

In addressing research question 1, the participants provided their own description or working definition of what these strengths involve and contextualized it with reference to the reality of the AEP. They also indicated how the presence of these three strengths could not only assist in AEPs’ successful adjustment in the host country, but also contribute to greater well-being. Perspectives on Curiosity, Gratitude and Hope involves the elements and time frame participants ascribe to these strengths.

### Elements of Strengths

Participants pointed out that the strength of *Curiosity* encompasses an open-mindedness and excitement about experiencing new things and exploring new possibilities. Participant 2 suggested that “curiosity can encourage a person to learn and think in different ways” (Participant 2; Quote 3). Participants highlighted that not only does curiosity encompass inquisition about all things new, it also promotes a sense of adventure and exploration, which clearly relates to AEPs’ decision to embark on expatriate assignment. According to Participant 3, “the positive rewards for curiosity is a wider world view and deeper and more meaningful experience of well-being” (Participant 3; Quote 18). Further, in the context of expatriation, Participant 3 proposed that “… [seeing] and subjectively [becoming] part of something new would seem more positive than being bored with the familiar” (Participant 3; Quote 17). Participant 2 concurred by stating: “Life is never boring for a curious person” (Participant 2; Quote 5).

The participating psychologists describe *Gratitude* in many different ways. One participant stated that gratitude “represents a willingness or ability” (Participant 1; Quote 5) to recognize what is good in life. Another participant proposed that gratitude refers to “a general state of thankfulness and appreciation” (Participant 2; Quote 13) and furthermore adds that it is a “life orientation that helps you notice and appreciate the world around you” (Quote 15). Further, one participant stated that gratitude makes it possible to recognize “good beyond [the] self” (Participant 4: Quote 11). Another participant agreed, adding that “the source of goodness lies at least partially outside themselves” (Participant 2; Quote 20), which “helps people connect to something larger than themselves as individuals — whether to other people, nature, or a Higher Power” (Quote 21). The reality is that AEPs are faced with numerous challenges prior to as well as during international relocation. In line with this, one of the practicing psychologists added: “I have found that some clients who have experienced, and are currently experiencing challenging times, seem to naturally move, or choose gratitude as a coping skill” (Participant 3; Quote 5).

As suggested by Participant 1, *Hope* “represents a motivation to look and expectation to find positive and beneficial details in one’s presence and future” (Participant 1; Quote 6).

Participant 2 and Participant 3 concurred, and stated that hope acts as a motivating factor to take positive actions and therefore “seems to create the platform from which they can launch their choices for their lives” (Participant 3; Quote 25). In addition, hope “develops people’s capacity for persistence and long-term efforts that makes them authors of their [own] lives” (Participant 2; Quote 27). This participant added that hope is “a vital coping resource against despair” (Participant 2: Quote 28b), as this strength prompts efforts to “seek improvement of unsatisfactory situations” (Quote 28a). It was evident that participating psychologists felt that when people have hopeful attitudes, they naturally trust and have faith that things will work out, despite their current reality of approaching the unknown. With regard to the role that hope plays in therapy with AEP clients, Participant 3 summarized it well, saying: “In my experience clients who have a clearly defined sense of future, that manifests in hope for a good future, are far more resilient than those who have vague ideas of what they want for their lives” (Participant 3: Quote 22).

### The Time Frame of Strengths

In this sub-theme, participants appeared to connect the strengths of Curiosity, Gratitude and Hope to either the past, present, future, or a combination of these time frames. This seems to inform how psychologists use them in therapy, which is brought into focus later on in this section. Participants agreed that *Curiosity* is situated in the present moment as well as in the future. Participant 2 stated that [curiosity] “helps you focus on the positive in the here and the now” (Participant 2; Quote 18). Participant 3 added that “the strength of curiosity drives certain people into making life decisions that others would avoid” (Participant 3; Quote 19). Participant 1 stated that the strength of *Gratitude* is used when reflecting on personal achievements in the past, and appreciating the present moment. Participant 4 concurred, adding that “drawing up a list of achievements” (Participant 4; Quote 61) can act as emotional leverage when facing other challenges. *Hope* on the other hand entails envisioning what is yet to come. Participant 3 stated: “Hope seems to bring thought together in a coherent picture, or narrative, or plan for ordering their expectations for the future” (Participant 3; Quote 23).

Participants’ perceptions of the strengths of Curiosity, Gratitude and Hope seemed to confirm the significance of these strengths and the role they play in the adaptation process of AEPs. Contextualized to the reality of the AEP, the reflections of participants suggest that each strength has a specific description or working definition and appears to be linked to a specific time frame.

### The Positive Impact of Strengths

This theme is based on the positive impact that strengths of Curiosity, Gratitude and Hope have on AEPs’ daily experiences as well as contributing to a sense of personal growth.

#### Positive Experiences of Strengths

Participants reported that positive experiences are associated with all three of the strengths mentioned above. One participant commented that “the positive experience of new things would soften the more severe challenges” (Participant 3; Quote 16), which suggests that the strength of Curiosity will have a positive effect on facing the daily challenges of adjusting to a foreign environment. International relocation signifies a traumatic life event that can often cause AEPs to feel uncertain, insecure, anxious and fearful (Botha, 2020). In line with this, another participant stated that Curiosity “acts as a positive counterweight to anxiety and fear” (Participant 2; Quote 11), suggesting that this strength plays a significant role in AEPs’ successful adaptation abroad. With respect to the positive experiences that Gratitude fosters, it was said that “gratitude helps people feel more positive emotions, relish good experiences, improve their health, deal with adversity, and build strong relationships” (Participant 2; Quote 22). Hope appears to be the strength that counteracts despair by fostering optimistic expectations that ultimately lead to meaningful experiences (Participant 2; Quote 25).

#### Personal Growth

Personal growth related to both Curiosity and Gratitude entailed making informed life decisions and overcoming fear and anxiety. One participant shared that “curiosity can open new doors and help [the AEP] to explore new possibilities and therefore make better informed choices” (Participant 2; Quote 6). Participants suggested that Gratitude leads to coping better in the face of challenges and being more grateful due to exposure to negative life events. Another participant emphasized the “importance of gratitude as a dimension for happiness, personal growth and success” (Participant 4; Quote 4). In accordance with Participant 4, Participant 3 affirmed that practicing gratitude in the AEP’s new environment, can result in “a possible growth experience through adversity and emotional memory recall - ‘I can remember that when I was grateful that I felt better’ or ‘let me find something to be grateful for and then experience gratitude intentionally’” (Participant 3; Quote 12). Although participants assert that practicing these strengths contribute to personal growth, it was also mentioned that the “growth mindset of a person enhances her well-being and resilience” (Participant 2; Quote 8). Simultaneously, “it is often in the evaluation of a real event that the deep experiences are realized” (Participant 3; Quote 41).

### Therapeutic Engagement and Goal-Setting

In addressing research question 2, therapeutic engagement refers to the aspects that practicing psychologists believe to be important when consulting with an AEP. Participants pointed out that a realistic initial assessment of context is imperative, and that it naturally leads to setting goals.

#### Assessment of Context

The first aspect of therapeutic engagement is an assessment of the AEP’s context. Participant 2 suggested that the starting point of the therapeutic process should be a “realistic initial assessment of personal resources and of external conditions and resources; (Participant 2; Quote 60). Participant 4 echoed that it is very important to know what the current context of the AEP is, and especially “how much autonomy or choice the individual has to work with” (Participant 4; Quote 48). According to this participant, this information “would then set the tone with regards to how prepared the AEP can be prior to arriving to their new post, i.e., using their forethought to create their future” (Participant 4; Quote 49). Many AEPs feel like they are “trailing” partners whose needs are not considered in the assignment contract and have to become stay-at-home parents in the host country as they had to give up their careers in their home country (Botha, 2020). Participant 4 highlighted the fact that psychologists should “encourage the AEP to find out what the benefits [of the assignment] are so that she can be prepared for the adventure ahead and set up some goals” (Participant 4; Quote 50).

#### Setting Goals

In addition to the initial assessment, psychologists highlighted the importance of taking the next step in therapy, and that is setting goals with AEPs. In line with the time frame of Curiosity, Gratitude and Hope, goal-setting starts with a deliberate focus on the present moment and current context of the AEP. Thus: “Actively embrace the here and the now, creating a sense of future that allows the family to enjoy the new adventure” (Participant 3; Quote 43). Participant 1 highlighted: “AEPs who naturally (or deliberately) incorporate gratitude, curiosity and hope into their lives are more likely to notice the things that are going well, [and] assign more meaning to them and possibly act in ways that confirm and elicit further such experiences” (Participant 1; Quote 10 and 11). Participant 3 agreed and added that “the deliberate focus on life content that leads to gratitude leaves people feeling less vulnerable in times of challenge” (Participant 3; Quote 3), which suggests that conscious optimism in the present moment leads to feeling stronger in times of adversity. However, participants do not proclaim that a deliberate focus on all things positive will do away with challenges. Rather, in highlighting “intentional and mindful focus on what is good, would minimize the focus on the obvious challenges” (Participant 3; Quote 29).

Envisioning the goal connects the present with the future, similar to strengths of Curiosity and Hope. This enables AEPs to increase their awareness of what they wish themselves to achieve in the future, which is “one of the best ways to have hope” (Participant 2; Quote 53). In the context of being an AEP in a foreign country, one participant suggested that “AEPs should develop the mindset of approaching their situation with the intention of discovering something useful that can help them to live a more fulfilling and interesting life” (Participant 2; Quote 37). Participants emphasized the fact that AEPs have their own hopes and dreams, that are just as relevant as the other family members’ hopes and dreams. Envisioning the goal is to be aware of “what [the AEP] is hoping for and what [the AEP] would like from the experience” (Participant 3; Quote 39).

### Facilitating AEPs’ Well-Being Through Strengths and Resilience

The final theme that emerged from participating practitioners’ reflections on the present model refers to the facilitation of AEPs’ well-being through strengths and resilience. This theme is therefore directly related to research question 2.

#### Cultivating Strengths and Resilience

As can be seen from the results above, participating psychologists’ reflection confirmed that the strengths of Curiosity, Gratitude and Hope as well as Resilience play vital roles in AEPs’ adaptation process and life abroad. They furthermore concurred that these elements or concepts can be cultivated and suggested a number of practical ways in which this could be achieved through informed intervention. The four psychologists who participated in the study appeared to have different therapeutic approaches and techniques that they utilize when consulting with AEPs. All of these techniques are aimed at fostering strengths, resilience and ultimately well-being of AEPs. Approaches included solution-focused techniques such as *best hopes, resource talk, preferred future description, scaling questions* and *invitation to notice details*. Other techniques that participants used were, among others, vision boards, journaling, jar of visions, reading and gaining knowledge, and meditation. In addition to the therapeutic techniques mentioned above, participants pointed out alternate ways in which to cultivate strengths of Curiosity, Gratitude, Hope as well as AEPs’ levels of resilience.

One participant stated that “*Curiosity* would probably the most obvious strength that I would expect to be present in this context [expatriation]” (Participant 3; Quote 14). According to another participant, “Curiosity can be cultivated by challenging clients to be open to new experiences and to remember to never stop learning and growing – which is the most exciting part of being alive” (Participant 2; Quote 36). Participant 2 furthermore suggested that psychologists can assist AEPs in cultivating curiosity in a number of ways, e.g., by motivating her to always ask questions and immerse herself in reading up on everything about the host country. Participant 3 agreed, and added that curiosity “can be enhanced by having sufficient information about where you are going, [and asking questions such as] ‘What will we experience that is unique to our new destination?’ [and] make a list of all the things that you want to do in your new city/country” (Participant 3; Quote 32). Once the AEP has arrived in the host country, one participant suggested that “families should be encouraged to participate in as many things as they can and to regularly talk to natives with the aim of trying to understand their world and viewpoints. Encourage families to form friendships. Demonstrating curiosity towards someone is a great way to build closeness with them” (Participant 2; Quote 38). In addition, the following: “The excitement and positive experience of travel and exposure to new cultures can of course just lead to easy and realistic gratitude. Just being grateful for the privilege and the experience could also be a logical explanation for the presence of gratitude” (Participant 3; Quote 13).

Participants proposed that *Gratitude* can be cultivated by introducing intentional, expressive practices to AEPs, such as rituals, mindfulness, deliberate focus on the positive, and also initiating self-care techniques. Participant 3 emphasized the importance of teaching AEPs that “intentional gratitude changes the emotions that they feel in the present and that it can be done wherever they are, the prerequisite being that they have to do it!” (Participant 3; Quote 30). Another participant echoed that gratitude can be cultivated, irrespective of where in the world they are, simply by “noticing details of x + 1 [recognizing the positive] happening in their lives” (Participant 1; Quote 16). Further, participants pointed out that gratitude can be cultivated within the AEP’s belief system, by prayer, meditation or a mindful focus on the feeling of gratitude. Another participant shared that she would “encourage the [AEP] to envision with hope … then sit in the gratitude of the present moment, get comfortable with the unfamiliar as the new knowledge the [AEP] gains in the unfamiliar will help her discover the ‘how to’s’” (Participant 4; Quote 63).

*Hope* can be cultivated by utilizing this strength as a personal resource or coping mechanism. Participants pointed out that most AEPs are able to find hope in something and that it is of great importance to “explore their best hopes from the expatriate assignment in detail” (Participant 1; Quote 11). Cultivating hope seems to correlate with goal-setting, as described in Theme 2. This is further highlighted by Participant 2, stating that “being able to see how the steps you are taking will lead to desired change is critical to having hope” (Participant 2; Quote 48). However, participants maintained that “in order to have hope, it is important to make sure that the vision the AEP has for herself is realistic. If not, it may cause hopelessness” (Participant 2; Quote 51). Other ways that appeared to cultivate hope, were to motivate AEPs to “practice mindfulness while doing acts of kindness and in your everyday life” (Participant 2; Quote 57), turn to their faith, and also spend time with people who have been through a similar experience.

There was only one participant who mentioned how the psychologist can assist in promoting the AEPs’ levels of resilience, stating: “For bounce back ability I get them to draw up a list of achievements that they can leverage off emotionally when faced with a challenge” (Participant 4; Quote 61). This example suggests that by shifting the focus on the positive, the AEP will feel stronger.

In addition to the above-mentioned suggestions regarding therapeutic strategies for the enhancement of these strengths in AEPs and thereby increasing their adaptability to the expatriate context, participating psychologists identified two important aspects. These included the important role of organizational support and the support received from other important interpersonal and community relationships.

#### Organizational Support and Other Supportive Relationships

In an attempt to help facilitate AEPs’ well-being, participants referred to the role that organizational support and other supportive relationships play.

As previously mentioned, the AEP plays a pivotal role in determining whether the international assignment is successful or not. It is a known fact that expatriate failure has a detrimental effect on cost-to-company. Participants expressed the importance for organizations (henceforth referred to as MNCs) to offer psychological support to AEPs prior to expatriation as well as during the period abroad. However, participants however pointed out that there appears to be a lack of, or insufficient support being offered to AEPs. One of the practicing psychologists who participated in the current study had been an AEP until 2018. She stated the following: “I’m told that in the past there was a person at head-office that tracked and kept up to date on each expatriate and their family. This individual had an in-depth knowledge of each family and their needs and I’m told offered invaluable support to the spouses. Over time this person was either retired or made redundant. I suspect that this individual’s role was merged into a general department and/or left to in-country personnel to deal with” (Participant 4; Quote 41). In addition, “I’ve never really received any company AEP type support” (Participant 4; Quote 42).

She furthermore pointed out that the contract of employment should be taken into account, stating that “[contractual information] is relevant though as it affects the AEP’s experience” (Participant 4; Quote 27). According to this participant, contractual elements that appear to be important to AEPs included housing; transport; job security of the employee; living expenses; medical aid; tax deductions; security; schooling and opportunities such as studying, working or volunteering in the host country (Participant 4; Quotes 29–38). Participant 3 agreed and suggested that all of the above should be considered prior to expatriation, to “avoid leaving the post as an alternative to resolving the crisis there” (Participant 3; Quote 45).

Participants pointed out that there are other supportive relationships that also contribute to the facilitation of AEPs’ well-being, such as shared AEP experiences, family relationships and building networks in the host country. This seems to correlate with the findings in the previous phase of this study (Botha, 2020). One participant stated that “the community becomes the support network that [AEPs] would have had back home” (Participant 4; Quote 44). Participants also emphasized the value of joining various expatriate groups in the host country and on social media. It appears as though fellow AEPs can motivate, empower and support one another (Participant 4; Quote 43). Most participants emphasized the importance of the family spending time together in the host country (Participant 2; Quote 38).

## Discussion

### Outline of Results

The practicing psychologists’ reflections and insights based on their professional experience highlighted the pivotal role that strengths of Curiosity, Gratitude and Hope and personal levels of resilience play in not only the AEPs’ adjustment process abroad, but also on their mental health. It is clear from their reflections that each strength has a specific role to play within the context of expatriation and is connected to a specific time frame. Where Curiosity was situated in the present moment and in the future, Gratitude was used when reflecting on achievements in the past and appreciating the present moment. Hope entailed envisioning what is yet to come, and was thus associated with a futuristic time frame. As mentioned in the results section, when considering the time frame connected to the above-mentioned strengths, participating psychologists pointed out that interventions had to start with a deliberate focus on the present moment, i.e., the current context of the AEP. In practice, this would imply that by establishing the starting point of the intervention, the opportunity is created to reflect on previous achievements, ascribe meaning to them in the present moment and set goals for themselves (as AEPs). By reflecting on previous achievements and ascribing meaning to them, the strength of gratitude is used, whereas setting goals incorporate strengths of curiosity and hope.

In turn, an increased awareness of what AEPs’ perceived as personal character strengths as well as challenges they successfully faced in the past, resulted in personal growth. This affirms that strengths can be cultivated and that the enhancement of strengths will have a positive effect on AEPs’ levels of resilience and overall well-being. The findings of this study indicate that not many psychologists deliberately focus on the enhancement of resilience. Panter-Brick and Leckman (2013) describe resilience as “the process of harnessing biological, psychosocial, structural, and cultural resources to sustain well-being” (p. 333). As fellow psychologists we have to take note and challenge ourselves to explore ways to use our clients’ unique resources with the purpose of enhancing resilience and ultimately increase well-being. Facilitating the strengths of curiosity, gratitude and hope seems to be especially relevant within the context of expatriation.

In addition to the enrichment of strengths and resilience to ultimately affect mental health, organizational support along with other supportive relationships (e.g., shared AEP experiences, family relationships and building networks in the host country) were considered as key elements in AEPs’ adaptation process and their mental health abroad. Furthermore, the findings of this study indicate that MNCs could experience better business outcomes through particular AEP supportive interventions, as employee’s performance at work is likely to improve when he receives moral support from the AEP and his family (Lauring and Selmer, 2010). This implies that supportive interventions for AEPs are of great importance, but also that it can possibly be an easy and cost- effective way for MNCs to increase their employees’ productivity and to improve the company’s financial gain. Once again, the relevance of AEPs’ mental health is affirmed. To be able to support the working partner, the AEP needs to feel strong.

### Practical Implications

As mentioned earlier, the AEP plays a fundamental role in not only the decision-making process prior to relocation, but also during the period abroad. In order to ensure successful expatriate assignments, it is of utmost importance that MNCs consider the needs of AEPs in the contract between the company and the working partner and that they offer them the appropriate psychological guidance and support while abroad. The challenges that MNCs, the working partner, the AEP and the family face during expatriation cannot be underestimated. The findings of an international study done by Lazarova et al. (2015) indicated that AEPs felt that “the support they received was largely inadequate (p. 9). To our knowledge, only one study has been done exploring the preparation, support and training requirements of South African expatriates, their spouses and families (Vögel et al., 2008). More than 10 years later, the results of the above-mentioned study are still relevant, as South African AEPs neither receive the preparation nor the psychological support they need. Psychological guidance and support can be provided by psychologists who regularly consult with working partners, AEPs and their families. As mentioned above, it is important that psychologists start with a thorough assessment of the current context of the AEP, where after personal expectations and goals can be put forward. By creating a solid starting point for the therapeutic intervention, the foundation is laid to incorporate strengths, enhance resilience and increase well-being.

### Limitations and Recommendations

It is important to note a few important limitations of this study. First, the sample group was relatively small (*n* = 4). Although the number was offset by the fact that there was representation from all three registration categories (i.e., clinical counselling and industrial psychologists) that often work with expatriates, and the large degree of overlap between practitioners’ reflections indicated a degree of data saturation, the possibility exists that participants’ respective reviews might not be as comprehensive as a larger sample groups’ review would have been. Second, while not necessarily a limitation, it should be noted that only practicing psychologists were included in the sample group, thus excluding human resource personnel of the MNC, business coaches or other counsellors. Insights from the MNC and other counsellors were therefore not included in the study and they may have provided valuable insight. Another possible limitation was that a simplified version of the path model (see Figure 2) was provided to participants, and this model did not include the strength of Humility that appeared to be negatively associated with well-being (phase 1). Despite the negative association between Humility and well-being, the researchers wanted to explore constructs that contribute to greater mental health.

It can be proposed that this study be expanded by including influential role players and obtaining insight from a human resource perspective. Our results clearly suggest that MNCs consciously include AEPs in conversations and decision-making prior to relocation, and that they would benefit from being mindful of not only the factors that could have a negative effect on AEPs levels of resilience or well-being abroad, but also of the aspects that could potentially contribute to greater mental health. It is thus proposed that proactive attention to AEPs’ mental health will benefit all parties involved, including the AEP, the working partner (expatriate), their children (if applicable). It is a well-established fact that the happiness or contentment of AEPs is a determining factor in the success or failure of the expatriate assignment. Perhaps most importantly from a business point of view, and especially within a context of an ever-increasing number of expatriate assignments, the mental health of AEPs should be regarded as top priority.

## Conclusion

The aim of the current study was to qualitatively review a proposed quantitative model with a panel of practicing psychologists who provide counselling services for South African expatriates and accompanying expatriate partners (AEPs). The objective was firstly to ascertain why these participants thought strengths of Gratitude, Curiosity and Hope were positively associated with AEPs’ personal levels of resilience and self-perceived well-being, and secondly, the study aimed to determine how these participants would, from their experience in working with AEPs, could potentially cultivate these strengths and AEPs’ resilience in therapy, and ultimately facilitate greater well-being and successful adjustment abroad. Participants confirmed that within the context of expatriation, all three strengths play important roles in the AEPs’ resilience and well-being abroad. The process of expatriation denotes change and embarking into the unknown. The strength of Curiosity is fueled by change and the exposure to new experiences. Gratitude referred to the ability to recognize the goodness in life, and was also described as a coping skill, especially during challenging times. Further, it was evident from participants’ reflections that people who practice Hope, naturally trust and have faith that things will work out, despite their current reality of approaching the unknown. Participants furthermore pointed out that these strengths were characterized by specific time frames, connecting the AEP either to the past, the present, the future or a combination of these. As mentioned in the results section, the participants appear to have different therapeutic approaches and techniques aimed at fostering strengths, resilience and ultimately well-being of AEPs. However, it was evident that practical strength-based techniques were incorporated into all four participants’ reflections. This study highlights the important role that both MNCs and psychologists should play in ensuring successful adaptation of AEPs. By providing AEPs with sufficient guidance and psychological support, it is proposed that the practical therapeutic techniques and approaches suggested in this study possibly will contribute to the success rate of expatriate assignments.

## Author’s Note

This article served as a partial requirement for the TB’s PhD degree. JP was the promotor and KB the co-promotor.

## Data Availability Statement

The raw data supporting the conclusions of this article will be made available by the authors, without undue reservation.

## Ethics Statement

The studies involving human participants were reviewed and approved by Health Research Ethics Committee (HREC), North-West University (Potchefstroom Campus), South Africa. Ethics number: NWU-00362-16-A1. The participants provided their written informed consent to participate in this study.

## Author Contributions

TB was responsible for setting up the research design, writing the literature review, as well as the process of data collection and data analysis. JP and KB guided the research process, edited and co-authored this article. All authors contributed to the article and approved the submitted version.

## Conflict of Interest

The authors declare that the research was conducted in the absence of any commercial or financial relationships that could be construed as a potential conflict of interest.

## Publisher’s Note

All claims expressed in this article are solely those of the authors and do not necessarily represent those of their affiliated organizations, or those of the publisher, the editors and the reviewers. Any product that may be evaluated in this article, or claim that may be made by its manufacturer, is not guaranteed or endorsed by the publisher.

## Footnotes

^1^“She” or “her” are used as personal pronouns referring to the AEP. The authors’ intent is non-sexist.

## Abbreviations

AEP: Accompanying Expatriate Partner
MNC: Multinational Company.
